# Perspective—Advances in Voltammetric Methods for the Measurement of Biomolecules

**DOI:** 10.1149/2754-2726/ad3c4f

**Published:** 2024-04-18

**Authors:** Nadiah Alyamni, Jandro L. Abot, Alexander G. Zestos

**Affiliations:** 1 Department of Biomedical Engineering, The Catholic University of America, Washington, DC, 20064, United States of America; 2 Department of Chemistry, American University, Washington, D.C. 20016, United States of America; 3 Department of Mechanical Engineering, The Catholic University of America, Washington, DC, 20064, United States of America

**Keywords:** Sensors, Electroanalytical electrochemistry, Graphene, Nanotubes

## Abstract

Voltammetry is a powerful electroanalytical tool that makes fast, real-time measurements of neurotransmitters and other molecules. Electroanalytical methods like cyclic, pulse, and stripping voltammetry are useful for qualitative and quantitative examination. Neurochemical sensing has been enhanced using carbon-based electrodes and waveform modification methods that improve sensitivity and stability of electrode performance. Voltammetry has revolutionized neurochemical monitoring by providing real-time information on neurotransmitter dynamics for neurochemical studies. Selectivity and electrode fouling remain issues for biomolecule detection, but recent advances promise new methods of analysis for other applications to enhance spatiotemporal resolution, sensitivity, selectivity, and other important considerations.

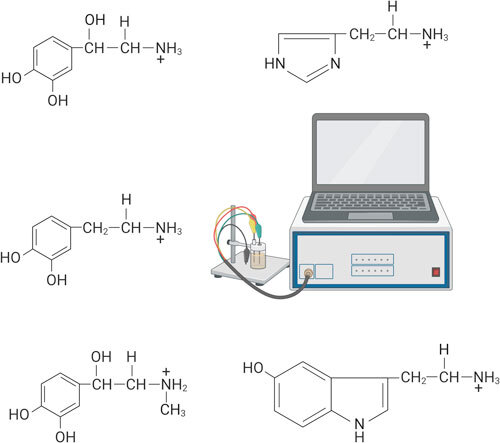

## Highlights

- Recent advances have been made in the voltammetric detection of biomolecules.
- Methods of measurement include cyclic, differential pulse, and square wave voltammetry.
- Carbon based electrodes are used for high biocompatibility and spatiotemporal resolution.
- New waveforms account for oxidation potentials and electron transfer kinetics of molecules.
- Novel electrode materials and waveforms need to be examined for challenges in measurement.

Voltammetry is a versatile analytical tool for studying electrochemical reactions in many materials. This technique has left a lasting effect on pharmacy, medicinal chemistry, biochemistry, and the life sciences.^1^ It offers valuable insights into electron and ion transfer processes, as well as kinetic and mechanistic aspects through straightforward experiments, thanks to its cost-effective instrumentation.^1^ The application of a voltage changes the sensor surface to intersect with the molecules’ redox reactions and current generation at specified energy levels. This technique requires a customized waveform. The voltammogram, is a current-vs-potential graph, and is a unique fingerprint for analyte identification and quantification.^2^ Heyrovsky received the 1959 Nobel Prize in chemistry for developing polarography in 1922.^3^ Early voltammetric methods were not applicable for everyday analytical applications due to their lack of chemical versatility. Improvements were made with respect to sensitivity and provided more analytical methods for voltammetric detection.^4^

Voltammetric methods measure electrochemical cell current by applying potential or voltage (*E*) to an electrode. As applied potential changes, current is carefully examined and changes over time according to the scan rate. Many benefits demonstrate voltammetric methods analytical strength. They detect inorganic and organic chemicals from 1 pM to 100 mM with high sensitivity and a broad linear concentration range. Voltammetry analyzes solvents, electrolytes, temperature, and time. It can test multiple analytes simultaneously, improving its analytical capabilities.^4^ For qualitative and quantitative electroactive species analysis, linear sweep voltammetry (LSV), cyclic voltammetry (CV), pulse voltammetry (PV), anodic stripping voltammetry (ASV), and chronoamperometry (CA) are essential. They offer promising alternatives to spectrometric or chromatographic procedures UV–vis absorption, fluorescence, IR absorption, NMR, and mass spectrometry for analyte detection.^5^ As such, they provide more in-depth quantification and qualification capabilities, along with higher sensitivities for electro-active species.^5^ These techniques are discussed in detail in the subsequent sections of this paper.

Voltammetry is a versatile biosensing method due to its high sensitivity, fast detection, and precise measurement capabilities. This adaptable methodology targets the electrochemical properties of analytes and quantifies sample attributes using diverse techniques.^6^ Here, we review recent voltammetric techniques, with an emphasis on how electrode and waveform development improve in vivo analyte discrimination and co-detection. We propose that by observing these characteristics, we can better understand the dynamic nature of voltammetry and its importance in analytical chemistry.

## Current Status

### Voltammetry strategies: pulse methods

Pulse-voltammetric methods are preferred for analytical measurements due to their high sensitivity. All pulse approaches rely on charging and faradaic currents’ unique behaviors after a potential step, or “pulse.” The charging current decays exponentially, but the faradaic current, decreases according to 1/(time)^½^ especially in diffusion-controlled current according to the Cottrell equation (Eq. 1)^7^ which describes the relationship between the current (*i*) flowing through an electrode and the rate of diffusion (*D*) of the analyte to the electrode surface. It elucidates how the current is directly proportional to the square root of time (*t*) and the concentration (*C*) of the electroactive species. The charging decays faster than the faradaic current and hence becomes negligible at five times the electrochemical cell’s time rate constant (*R_u_C_dl_,* which can be microseconds to milliseconds). At this stage, the measured current is only faradaic. Thus, measuring the current after a potential pulse distinguishes faradaic and charging currents.^8^

The key is that the charging current decays much faster than the faradaic current. By measuring the current at the end of the pulse after the charging current has decayed, but before the faradaic current has substantially decreased, the contribution from the charging current is minimized and the measured current is primarily faradaic. This allows pulse methods to discriminate faradaic and charging currents for improved analytical signals.

\begin{eqnarray*}{i}_{c}={nFAC}{D}^{1/2}{\pi }^{1/2}{t}^{1/2}\end{eqnarray*}

Equation 1: Cottrell equation where *i_c_* is the faradaic current, n is the number of electrons transferred, *F* is the Faraday constant, *A* is the electrode area, *C* is the bulk concentration of the electroactive species, *D* is the diffusion coefficient, and *t* is time.

Normal Pulse Voltammetry (NPV), initially referred to as Normal Pulse Polarography (NPP), was developed through the pioneering work of Barker and colleagues.^9^ This technique was originally designed for the Dropping Mercury Electrode (DME), where the potential pulse is given at the end of the drop and the current depends on pulse duration and drop longevity. Short-term measurements are taken to eliminate the capacitance component and improve sensitivity. Potential pulses with increasing amplitudes are used in NPV. Current measurements toward the conclusion of each pulse allow charging current to dissipate. The voltammogram shows sampled current on the Y-axis and applied potential on the *X*-axis.^10^

Similar to normal pulse voltammetry (NPV), differential pulse voltammetry (DPV) also applies discrete potential pulses during the scan. However, while NPV measures the current before and after each pulse, DPV measures the current difference induced specifically by each potential pulse. Unlike NPV, each potential pulse is fixed, and has a tiny amplitude (10–100 mV) and overlays a progressively changing base potential. One study explored the use of DPV for the detection of lead and cadmium in environmental samples, demonstrating its applicability in environmental monitoring.^11^ In another study, the authors developed a biosensor system that effectively detects epinephrine using a poly-thiophene derivative and tyrosinase as a biorecognition element, with the differential pulse voltammetry technique illustrating enhanced sensitivity, stability, and surface coverage.^12^ Current is measured twice, before and after each pulse. Charge current is a relatively continuous baseline on the voltammogram, boosting background separation and lowering detection limits. High sensitivity makes DPV the best electrochemical method for trace detection of inorganic and medically relevant compounds.^13,14^

Square-wave voltammetry (SWV) increases analytical performance and provides mechanistic and kinetic insights into electrochemical processes. Its high sensitivity and background current filtering are advantageous. SWV contains quick scan speeds that let the DME move through the voltage range in one drop during polarography. This velocity, computerized control, and signal averaging enable repetitive trials and increase the signal-to-noise-ratio (SNR).^15^ Recent work has introduced continuous SWV (cSWV) which collects current continuously during the SWV waveform to extract multiple voltammograms from a single scan.^16^ This shows promise for conformation switching biosensors by rapidly assessing frequency dependence.^16^

SWV simulates charging current at pulse end to distinguish it from faradaic current. Net current is the difference between forward and reverse currents from these two sample sites. It has a faster detection time than DPV despite identical sensitivities. SWV is prominent in fundamental research and biological chemical analysis because it analyzes reversible or quasi-reversible electrode reactions.^17,18^ DPV conversely excels at analyzing irreversible electrochemical reactions that benefit less from varying frequency.^19^ The differences arise from SWV’s potential waveform that samples charging and faradaic current at different points in the pulse to reject the charging contribution.^19^

### Preconcentration and stripping techniques

Electroanalysis can utilize sample preconcentration and stripping to enhance sensitivity for trace element and compound analysis. Analytes are often preconcentrated on an electrode surface and released or stripped to enable sensitive and selective measurement.^20^ Three types of these techniques, each tailored to certain analytical needs and chemistries, aim for precise readings. Anodic Stripping Voltammetry (ASV), a popular trace metal detection method, has a part-per-trillion detection limit. This increased sensitivity is complemented with affordable gear that can measure four to six trace elements. ASV concentrates sample solution metal ions on a Mercury electrode for a set duration using a negative potential.^21^ The voltage is then scanned positively using linear sweep voltammetry (LSV) to oxidize and strip each metal from the surface, producing a peak current proportional to its concentrated amount. For example, trace metals can be electroplated onto a carbon electrode surface at a negative potential.^22^ This deposits a metal amalgam on the carbon surface and concentrates the metals from the dilute sample solution. Applying an increasingly positive potential then oxidizes each metal species at its characteristic potential, stripping it from the surface as a cation. This stripping produces a peak current for each metal that depends on its concentrated amount and original sample solution concentration.^22^

Real-Time Subsecond Voltammetric Analysis of lead (Pb) in aqueous environmental samples has been achieved with anodic stripping. This technique is essential for assessing and modeling Pb movement during such events, aiding environmental damage reduction.^23^ A unique adsorption-controlled electrochemical process of copper has been discovered by studying the thermodynamics of surface copper adsorption and the electrochemical features of copper deposition onto carbon-fiber microelectrodes (CFMEs).^24^ Recent work has also shown electro-precipitation of cocaine followed by voltammetric oxidation can provide selective cocaine detection in low conductivity media like tap water.^25^ This takes advantage of the pH-dependent solubility of cocaine by driving water reduction at the electrode surface to increase local pH and precipitate cocaine base. Oxidizing this precipitate then provides a selective voltammetric signal.

Cathodic Stripping Voltammetry (CSV) has been utilized to measure insoluble mercurous salts. When given a strong positive voltage, a Mercury electrode in such a solution generates an insoluble layer. An effective negative potential scan “strips” this deposited film into solution. CSV detects halides, selenide, sulfide, and oxyanions.^4^ The cathodic stripping technique is used in organic and medicinal chemistry for analyzing a variety of pharmacological substances.^22^ CSV also helps determine reactive Arsenic (As(III)) at a vibrating gold microwire electrode. Adsorptive deposition of Arsenic Hydroxide (As(OH)_3_) detects As(III), followed by a potential scan to determine the reduction current from As(III) to As(0).^26^ CSV can also evaluate natural water humic compounds.^27^ In another work, catalytic cathodic stripping voltammetry utilized iron-humic substances (IHS) complexes to adsorb on a Mercury drop electrode at natural pH.^28^ This method was validated by establishing the voltammetric response (peak potential and sensitivity) for IHS in natural water samples using a fulvic acid standard.^29^

Adsorptive Stripping Voltammetry (AdSV) is similar to anodic/cathodic stripping, however, instead of electrolysis, the preconcentration concentrates analytes by adsorption on electrodes or chemically modified electrodes. Voltammetric methods such as DPV or SWV measure negative or positive adsorbed species. This approach produces peak-shaped voltammetric response with analyte concentration-dependent amplitude. Heme, chlorpromazine, codeine, and cocaine can be detected at micromolar and nanomolar levels by AdSV.^4^ Various investigations show that drug adsorption onto the electrode surface increases sensitivity.^30^ By carefully analyzing operating factors, these methods attain very low detection limits. These approaches are cheaper and faster than differential pulse adsorptive stripping voltammetry (DPAdSV) and square wave adsorptive stripping voltammetry (SWAdSV).^31^

### Cyclic voltammetry

**Figure 1.**
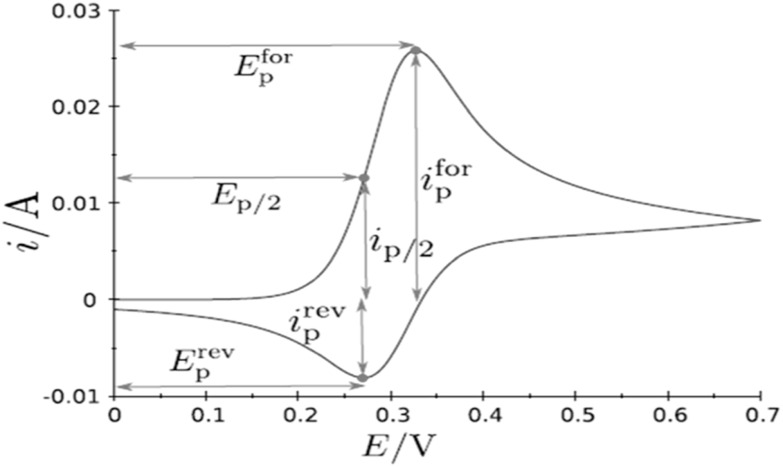
A typical cyclic voltammogram showing respective oxidation and reduction potentials and currents. The voltage is scannedfrom 0 V to 0.7 V and back. Reproduced with permission.^32^

**Figure 2.**
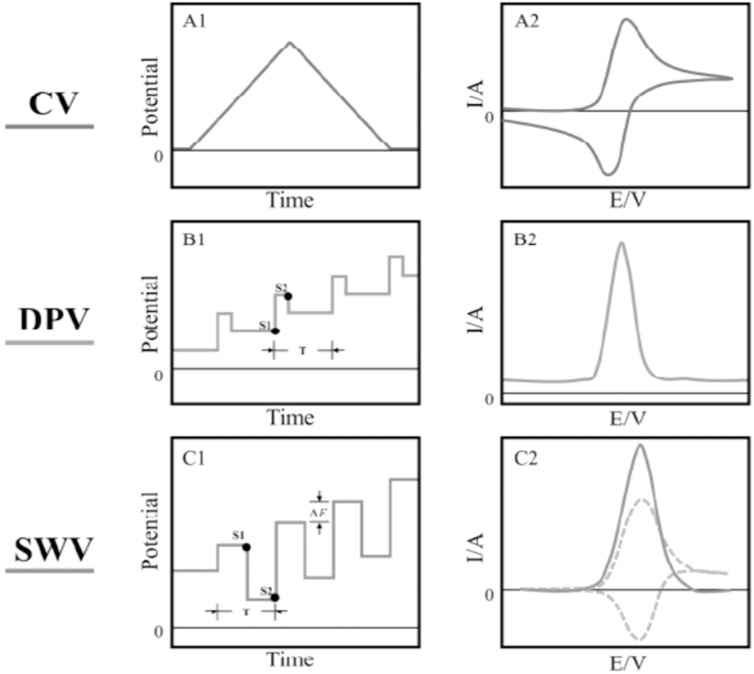
Waveforms and responses for electroanalytical techniques. (A1) Applied cyclic potential waveform displaying voltage over time. (A2) The resulting CV voltammogram, which plots measured current vs voltage. (B) DPV (Differential Pulse Voltammetry). (B1) A differential pulse potential waveform with T as the pulse period and S1 and S2 as current sampling points. (B2) The final DPV voltammogram. SWV (Square Wave Voltammetry). (C1) A square wave potential waveform is applied, where *E* is the pulse increment and T is the pulse period. (C2) The resulting SWV voltammogram (dashed lines) is made up of forward (anodic) and backward (cathodic) components, the net difference of which yields the overall response (solid line). Reproduced with permission.^6^

Cyclic voltammetry (CV) is an important electroanalytical technique utilized in several disciplines. It examines redox processes, reaction intermediates, and reaction product stability.^33,34^ CV measurements typically use a three-terminal cell with a working electrode (WE), counter electrode (CE), and reference electrode (RE). The voltammogram shows sampled current on the *Y*-axis and applied potential on the *X*-axis (Fig. 1).^10^ Through the working electrode, the sample’s current response to a sweeping voltage potential is monitored. CV is very sensitive to low-molecular-weight antioxidants (LMWA), which helps combat oxidative stress.^35^ Fast-Scan Cyclic Voltammetry (FSCV) is a significant improvement over standard cyclic voltammetry (CV), a classical electrochemical method for analyzing electroactive species’ redox processes by potential sweeping. FSCV was developed by Millar and colleagues in 1979 and popularized by the Wightman group.^36^ Savéant and Amatore expanded FSCV theory and instrumentation, especially at the ultramicroelectrode size.^37^ With FSCV, a two-electrode configuration is often employed, with the understanding that such a setup is adequate for experiments involving the passage of low currents. FSCV operates at scan rates of 100 Volts per second or faster, unlike standard CV, which operates at tens to hundreds of millivolts per second. This gives FSCV excellent temporal resolution and sensitivity, making it ideal for recording and measuring neurotransmitter dynamics in vivo.^38^ Figure 2 illustrates the waveforms and corresponding current responses for three electroanalytical techniques: Cyclic Voltammetry (CV), Differential Pulse Voltammetry (DPV), and Square Wave Voltammetry (SWV), used for probing the redox characteristics of analytes. For CV (Fig. 2A1), a triangular voltage waveform sweeps back and forth between two limits, generating a characteristic cyclic voltammogram (Fig. 2A2) with peaks representing redox events. DPV (Fig. 2B1) employs a staircase waveform with superimposed voltage pulses, enhancing sensitivity and resolution by measuring the current difference before and after each pulse, reflected in sharp peaks on the voltammogram (Fig. 2B2). SWV (Fig. 2C1) applies a square waveform, altering between forward and reverse pulses, with the current response (Fig. 2C2) capturing the difference at each step, thus enabling the detection of distinct redox-active species with heightened sensitivity. These methods are pivotal in electrochemical analysis, providing insights into the kinetics and mechanisms of electrochemical reactions.

### Waveform strategies for in vivo analyte detection

Dynamic voltammetry requires precise potential control with a pre-defined waveform. Early analog devices used linear scans to change preset values continuously. Software-configurable modulation and waveform characteristics in computer-controlled instruments give operators flexibility. Commonly utilized waveforms are linear scan (LS), differential pulse (DP), triangle, and square ones.^39^ To switch electrode potentials, cyclic voltammetry (CV) uses a staircase triangle potential waveform and linear potential scan. Using a symmetrical square wave (SW) pulse superimposed on a staircase waveform, Square-Wave Voltammetry (SWV) matches the staircase step with the forward square pulse.^40^ Many new waveforms have improved and diversified sweep-wave voltammetry.^41^ Other studies have used waveforms with varying scan speeds in one sweeping direction, and that includes the triangle sweeping waveform. This invention improves species-electrode interactions and redox kinetics through the manipulation of electron transfer at the surface of the electrode.^42^

A tailored waveform by the Sombers laboratory^43^ predicts and subtracts drift in dopamine, adenosine, hydrogen peroxide (H_2_O_2_), and pH readings. They also used sweep waveforms to identify methionine-enkephalin (M-ENK), leucine-enkephalin (Leu-ENK), and H_2_O_2_, demonstrating their analytical versatility.^44^ Moreover, Fast Cyclic Square-Wave Voltammetry, developed by Park and colleagues, is also a groundbreaking electrochemical method.^45^ This novel method integrates Large-Amplitude Cyclic Square-Wave Voltammetry (CSWV) with background subtraction, improving voltammetric analysis by enhancing selectivity and sensitivity in a combined two-dimensional voltammogram.^45^ Even though FSCV utilizes the triangular waveforms, modifications to this waveform have enhanced analyte sensitivity and stability at high scan rates.

The now conventional “Dopamine waveform” was developed by altering the switching potential from 1.0 V to 1.3 V, which was shown to increase sensitivity by renewing, etching, and regenerating the electrode surface.^46,47^ “Sawhorse” waveforms with an extended hold at 1.3 V enhance CFME sensitivity at scan speeds of ≥1000 V s^−1^.^48^ To measure opioid neuropeptide M-ENK, which comprises electroactive amino acids like tyrosine and methionine, the Sombers group developed a modified sawhorse waveform with multiple scan rates to optimize the oxidation of tyrosine.^49^ Other studies have used this waveform to differentiate dopamine and other monoamines from other tyrosine-containing neuropeptides such as oxytocin based on the varied oxidation potentials of the molecules.^15^ When applied with MSW (multiple scan rates) on CFMEs, FSCV also provides information on real-time oxytocin effects on rat brain slices, exposing its impact on physiology and social behavior.^15,50^ The Jackson (serotonin) N-shaped waveform was also shown to be optimal for the detection of serotonin with FSCV.^51^ This waveform holds at a potential of +0.2 V to avoid serotonin fouling byproduct adsorption, and rapidly increases to 1.0 V at 1000 V s^−1^ to prevent fouling, and then scans down to −0.1 V during the reverse sweep to produce the cathodic peaks. These advances show further waveform technique refinement for voltammetric analyte detection.^46,52^ Table I illustrates commonly used voltammetric techniques employed in the electrochemical detection of analytes along with evaluation parameters.

**Table I.** Comparison of voltammetric techniques. A few voltammetric techniques are compared with respect to time of analysis, example waveforms, and limit of detection for certain analytes.

Techniques	Analysis time	Example waveforms	Limit of detection (LOD)	References
Fast Scan Cyclic Voltammetry (FSCV)	Subsecond time scale with a 400 V s^−1^ scan rate and a frequency of 10 Hz.	Sawhorse waveform	0.96 ± 0.08 nM in vitro for dopamine	53
		Triangular		
Linear Sweep Voltammetry (LSV)	Seconds to minutes timescale	Continuous cyclic	0.015 μM for urea	54
Differential Pulse Voltammetry (DPV)	Seconds timescale	Staircase	0.122 μM for catechol	55
Square Wave Voltammetry (SWV)	Subsecond timescale	Square	0.17 nM for dopamine	56

### Future needs and prospects

Advancements in voltammetric methods are crucial for in-depth neurochemical analysis, enabling the study of neurotransmitter dynamics and molecular activities in the nervous system. Techniques such as FSCV have been pivotal in capturing neurotransmission at rapid timescales, offering insights into phenomena like dopamine’s role in behavior. For instance, it was shown that subsecond midbrain dopamine release promotes cocaine-seeking in rats where dopamine release increased instantaneously open the application of a cue before self-administration of cocaine occurred.^57^ The near future necessitates a broader spectrum of analytical tools for precise and specific neurochemical dissection, potentially transforming our comprehension of synaptic functions and neuroplasticity. The challenge remains to accurately detect neurotransmitters within the brain’s complex biochemical milieu. Innovations in electrode design and surface modifications are expected to improve selectivity and sensitivity, facilitating the concurrent monitoring of various neurotransmitters and their by-products. Integration with genomics, proteomics, and computational neuroscience over the next decade will likely yield a comprehensive understanding of brain functionality, linking molecular actions to behavioral patterns and aiding in the development of targeted therapies for neuropsychiatric conditions. The use of artificial intelligence in analyzing neurochemical data promises to enhance the accuracy and applicability of voltammetric techniques significantly.

## Conclusions

Voltammetry, a robust analytical method, has significantly advanced in sensitivity and selectivity, enhancing biomedical research. This technique, through methods like cyclic and stripping voltammetry, has improved the detection and analysis of biomolecules, aiding in neurochemical monitoring. Progress in electrode materials, especially carbon-based ones, has boosted neurochemical sensing. Despite obstacles such as selectivity enhancement and fouling issues, ongoing innovations in waveforms and electrode adjustments promise further breakthroughs. For voltammetry to reach its full potential in biomedical analysis, addressing challenges like selectivity in complex media and developing antifouling materials is crucial. Future efforts will focus on refining temporal resolution and detection limits, paving the way for novel insights into biochemical processes and single-molecule measurements.
